# Advanced Preoperative Planning Techniques in the Management of Complex Proximal Humerus Fractures

**DOI:** 10.7759/cureus.51551

**Published:** 2024-01-02

**Authors:** Zaid Yasen, Andrew P Robinson, Hugo Woffenden

**Affiliations:** 1 Trauma and Orthopaedics, Royal Free London NHS Foundation Trust, London, GBR; 2 Trauma and Orthopaedics, Lewisham and Greenwich NHS Trust, London, GBR; 3 General Surgery, HMS Nelson Medical Centre, Ministry of Defence, London, GBR

**Keywords:** orthopaedic trauma, surgical simulation, 3d printing, cavst, proximal humerus fractures, pre-operative planning

## Abstract

This review evaluates the current literature on the recent advances of preoperative planning in the management of complex proximal humerus fractures (PHF). PHFs can pose a considerable challenge for orthopaedic surgeons due to their diversity in presentation and complexity. Poor preoperative planning can lead to prolonged operations, increased blood loss, higher risk of complications, and increased stress on the surgical team. Recent advances have seen the evolution of preoperative planning from conventional methods to computer-assisted virtual surgical technology (CAVST) and three-dimensional (3D) printing, which have been highlighted as transformative tools for improving preoperative planning and postoperative outcomes. CAVST allows the creation of 3D renderings of patient-specific anatomy, clearly demonstrating fracture patterns and facilitating detailed planning for arthroplasty or surgical fixation. The early studies show promising outcomes however the literature calls for more high-quality randomised controlled trials. Using 3D printing for high-fidelity simulation involving patient-specific physical models offers an immersive experience for surgical planning. Preoperative planning with 3D printing reduces operative time, blood loss and use of fluoroscopy. The technology’s potential to produce customisable surgical implants further improves its versatility. There is a need for a cost analysis for the use of these technologies within the orthopaedic field, particularly considering the high expense of 3D printing materials and extended hospital stays until the printed models are available. CAVST and 3D printing also show promising applications within high-fidelity simulation surgical training, with CAVST offering possibilities in virtual reality and haptic-enhanced simulations and 3D printing providing physical models for trainee surgeons to hone their skills. Moving forward, a reduction in the cost of 3D printing and the advancement of CAVST using artificial intelligence would lead to future improvement. In conclusion, preoperative planning supported by these innovative technologies will play a pivotal role in improving surgical outcomes and training for complex PHF cases.

## Introduction and background

Proximal humerus fractures (PHF) vary greatly in their presentation, complexity, and morphology; as such, they often represent a challenge to the orthopaedic surgeon in the planning and decision-making of optimal management [1].

Meticulous preoperative planning offers numerous benefits to the patient, the surgeon, and the theatre staff. This holds particularly true in orthopaedic trauma, where consideration of anatomical planes, surrounding structures, and end-result stability are of paramount importance [2]. A survey found that whilst nearly all orthopaedic consultants and trainees felt that preoperative planning was important, more than half would do so in routine planning [3]. Failing to plan for any procedure, especially a complex orthopaedic fracture fixation, can result in poor surgical outcomes, including longer operative time, increased blood loss, greater stress for the surgical team, and higher complication risks in the long and short term.

The process of surgical planning is not a new one but has been a consistently evolving field which has built on advancements in imaging technology. Prior to digital radiographs, surgeons would hand-trace over images on a physical plane film to delineate the anatomy of the fracture, and compare against a reversed image of the contralateral limb [1]. This, of course, provides a low-fidelity simulation and does not allow for three-dimensional (3D) visualisation or account for anatomical variation. As digital imaging was adopted and computed tomography (CT) became widely available, the nature of preoperative planning progressed. CT imaging provides greater delineation of fracture patterns and, as such, allows the surgeon a more robust step-by-step cognitive process in planning surgical technique [4].

Recent developments in the planning of complex fracture operative management have seen the advent of high-fidelity models that include computer-assisted virtual surgical simulation and 3D printing [5]. This review will describe the current state of surgical options in complex proximal humerus fractures, how recent innovations in preoperative planning are impacting their outcomes, and the future direction.

## Review

The PHF

Like with many fracture types, the proximal humerus sees a bimodal age distribution in the presentation of fracture with a noted sex discrepancy, presenting in younger males as a consequence of high-energy injuries, and in older females as a fragility fracture in osteoporotic bone following a fall on an outstretched arm from standing height.

The sites at greatest risk of fracture in the proximal humerus correspond with its anatomical bony landmarks. The anatomical neck marks the beginning of the articular surface proximally, whilst the surgical neck indicates the start of the humeral shaft distally. The greater and lesser tuberosities serve as rotator cuff muscle attachments and may be seen to be displaced in particular fracture patterns [2].

Classification

Classification is currently derived from clinical imaging using orthogonal X-rays and CT imaging [2], with MRI rarely being used unless there is significant soft tissue involvement.

The Neer classification (Table 1) groups PHFs based on the number of displaced bony landmarks mentioned above. They are categorised into one-, two-, three- or four-part fractures, with the latter two being defined as complex fractures [6].

**Table 1:** Neer Classification Categories and Descriptions Information source: Marongiu et al., 2020 [7]

Classification	Description	Notes
1 part	Surgical neck, anatomic neck, lesser tuberosity or greater tuberosity	Any fracture pattern with less than 1 cm displacement
2 part	Surgical neck, anatomic neck, lesser tuberosity or greater tuberosity	Fragments must be displaced by 1 cm
3 part	Surgical neck and greater tuberosity or surgical neck and lesser tuberosity	Fragments must be displaced by 1 cm
4 part	Surgical neck, lesser and greater tuberosities	Fragments must be displaced by 1 cm

Arbeitsgemeinschaft für Osteosynthesefragenbeing (AO) also categorises fractures into two-, three- and four-part fractures with further subgroups depending on the location of the displaced fracture [8].

Both of these systems show a deficiency in reliability as there are discrepancies in interobserver agreement on fracture pattern classification from imaging [7]. As such there is scope for 3D technologies to improve the rate of agreement amongst surgeons.

Management

The PHF may be managed conservatively or with a range of surgical options. Decisions on which management option to employ will be influenced by the patient's age, functional status, co-morbidities, fracture pattern, and bone stock.

Minimally displaced fractures are nearly always treated conservatively with a collar and cuff, elevating the wrist above the elbow to allow gravity to encourage natural alignment, along with physical rehabilitation. A patient aged over 65 years with co-morbidities is far more likely to have conservative treatment [9]. Notably, the ProFHER (PROximal Fracture of the Humerus: Evaluation by Randomisation) trial of 2015 advocates the use of conservative management in place of surgery even in three- and four-part fractures that are not associated with dislocation of the shoulder [10].

Surgical options for the patient include fixation or arthroplasty. Fixation may take place in the form of closed reduction percutaneous pinning (CRPP), open reduction internal fixation (ORIF), or intramedullary (IM) nailing. Older patients, those with poor bone quality, and those at risk of avascular necrosis are likely to undergo arthroplasty, which may be in the form of hemiarthroplasty which spares the supraspinatus as the primary abductor or reverse polarity arthroplasty in which the deltoid becomes the primary abductor [11]. Complication rates are comparable between the fixation methods, whilst arthroplasty has a lower complication rate with a reduced functional outcome [12]; as such, it is used more often in those over 65 years of age. Very little is set in stone in operation choice and a review of surgeons perspectives on PHF in the elderly found wide discrepancies in their risk/benefit perception of surgical management [13].

This overview of PHF management highlights the complexities of accurate diagnosis and selection of treatment modalities. Precise preoperative planning is therefore imperative to maximising surgical outcomes with this pathology.

Preoperative planning: current advancements

The introduction outlined the historical methods of pre-operative planning for trauma and orthopaedics. While the use of CT imaging has allowed the surgeon to more easily envision a realistic projection of the patient’s fracture, it was the advent of computer-assisted virtual surgical technology (CAVST) and 3D printing that truly enabled an interactive 3D solution to display the step-by-step techniques for the surgical team. Both are in the early stages of implementation into practice and display their advantages and disadvantages with various scopes for innovation in the field of orthopaedic preoperative planning. Aside from the obvious advantages of the interactive properties of these two technologies, they also benefit from recreating patient-specific anatomy, allowing confidence and clarity in the approach regarding nearby structures and fracture planes [14].

CAVST

CAVST builds 3D renderings of patient-specific anatomy and fracture patterns from MRI or CT digital imaging and communications in medicine (DICOM) data [15]. The surgeon can move fracture segments on the computer screen to identify the steps necessary for fracture reduction and fixation [16]. These systems have been shown to improve patient outcomes in multiple complex fracture types, including pelvic, hip and PHFs [17].

Numerous studies have assessed the benefits of CAVST in the preoperative planning stage of PHF regarding both fixation and arthroplasty. These have been used successfully, not only to allow the surgeon a chance to attempt virtual reconstruction prior to the operation itself but also to accurately predict the premorbid anatomy of the proximal humerus prior to fracture and allow calculation of the parameters required for successful reduction to normal anatomy [18-20].

While these studies have displayed encouraging results, there is a lack of high-quality randomised control trials (RCTs) in the use of CAVST in orthopaedics in general, let alone specifically in the PHF [19].

3D printing technology

Using the same principle of 3D reconstruction from thin-slice CT imaging, it is possible to produce anatomically accurate physical fracture models. This allows for incredibly high fidelity, hands-on experience in creating a bespoke surgical plan for the patient-specific anatomy of the fracture [21]. The surgeon can determine fixture size, orientation of screws, and any requirements for bone grafts. A physical model allows for proprioceptive input and haptic feedback in simulating a specific trauma operation [22,23].

It is therefore not surprising that a meta-analysis of 12 RCTs assessing the incorporation of 3D printing in preoperative planning versus conventional surgical planning found the former group to have significantly reduced operative time, blood loss, and fluoroscopy use along with faster time to fracture union and the rate of excellent outcomes [23]. A systematic review and meta-analysis that included multiple levels of evidence, along with a scoping review of RCTs reciprocates these findings [24,25].

**Figure 1:**
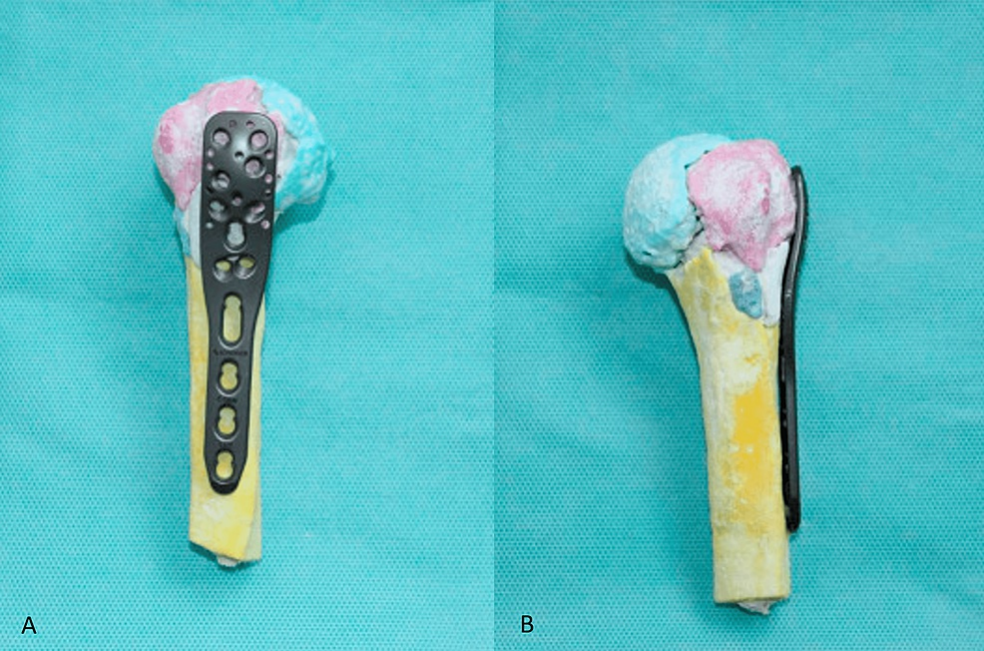
Lateral (A) and Posterior-anterior (B) Views of a 3D Synthesis Recreation of a Patient-Specific Proximal Humerus Fracture with Application of a Fixation Plate 3D: three-dimensional Image Source: You et al., 2016 [26]; Published with Permission. Copyright © 2016. Orthopaedics and Traumatology: Surgery and Research, published by Elsevier Masson SAS. All rights reserved.

A 2016 RCT by You et al. included in the above review specifically describes the use of 3D printing to synthesise patient-specific models (Figure 1) to aid the management of PHF, where the positive results of surgical outcomes in the 3D-printing group are congruent with the overall data from the meta-analysis [26]. The study highlights that surgeons reported a better understanding of the complex PHF pattern and could more easily devise an operative plan. A further important aspect to note is that doctor-patient communication was improved as the surgeon could more easily explain the intricacies of the operation with a real-life rendering of the patient's own fractured humerus.

A component of preoperative planning in PHF includes the correct diagnosis of fracture pattern to determine the optimal treatment modality. Earlier in this review, the disparity in the reliability of different classification systems is highlighted. A 2022 study by Cocco et al. found that the inter-rater agreement in the diagnosis and treatment indication for PHF between shoulder surgeons was greater using 3D printed models than CT imaging, although this was based on a small sample size [27]. The 2016 RCT by You et al. also reports a more accurate preoperative diagnosis of the fracture patterns [26].

Direct comparison of preoperative planning technologies in OHFs

 A 2018 retrospective study by Chen et al. compared the functional and radiographic outcomes in the surgical management of complex PHF based on the preoperative planning method [14]. Groups were divided into conventional methods, CAVST, and 3D printing. Both CAVST and 3D printing were superior to the conventional group regarding operative time, blood loss, and the amount of fluoroscopic imaging used. The clinician-assessed and patient-reported outcomes were also improved. There was no significant difference in radiographic outcomes with neck-shaft angle (NSA) and humeral head height (HHH) being measured.

The CAVST did hold the advantage of time over the 3D printing group. Hospital stay was significantly reduced due to the time to surgery being nearly double in the 3D printing group, whilst the preoperative planning time was over eight times more. There was no significant difference in hospital stay between the CAVST group and the conventional group. Interestingly, the CAVST group displayed a better correlation between the length of the screw chosen preoperatively compared with intraoperatively than the 3D printing group.

Implants

**Figure 2:**
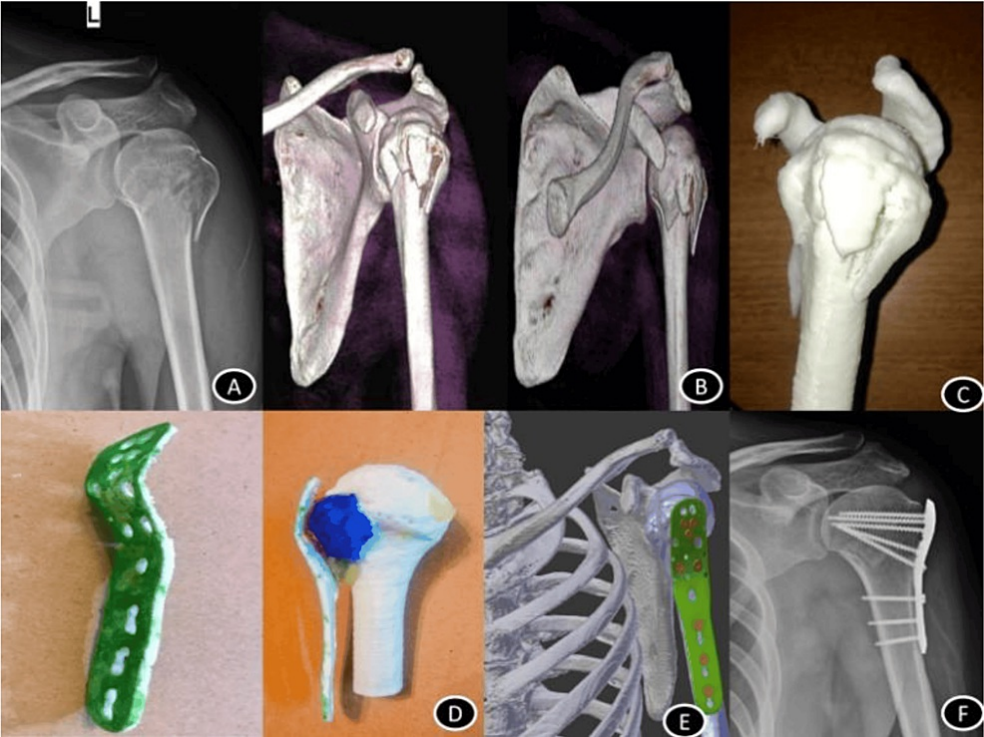
Steps of 3D Printing a Proximal Humerus Fixation Plate Image Source: Mishra et al., 2019 [28]; Open access under the CC BY-NC-ND license

The scope of 3D printing goes beyond recreating the patient’s anatomy. We now have the capacity for customisation of implants for arthroplasty as well as fixation plates, given that we can accurately render the patient’s premorbid anatomy. This allows the surgeon to trial the implant on a synthetic bone model (Figure 2), to then be sterilised and used again in theatre [28]. A 2019 study by Hu et al. found that the use of personalised prosthesis in reverse polarity shoulder arthroplasty resulted in improved shoulder function as well as a reduced rate of complication as compared to a conventional prosthesis [29].

Consideration of cost

There is a lack of literature assessing the overall cost per case in the use of both CAVST and 3D printing specifically in orthopaedic fracture management. Most studies pertaining to the cost analysis of these technologies are within the field of maxillofacial surgery [30]. Centres utilising 3D printing must take into account not only the cost of the physical printer but also the printing materials themselves [31]. A 2020 study evaluated the cost of in-house printing of mandibular models in facial trauma to be $1677.82/patient [32].

A further cost of 3D printing is the related extended hospital stay. On the other hand, some cost is recuperated with both technologies given the reduced operating time. This will likely not be a negligible sum, given that a 2016 study at St Mary’s Hospital in London calculated the cost of operating theatre use to be £24.77/minute [33].

Training potential

The benefits of 3D printing in preoperative planning for training purposes have already been highlighted and are significant. It provides a higher fidelity model of simulation with the incorporation of proprioceptive and haptic sensory input along with easy access to 360° visualisation of patient-specific anatomy. Given the high cost of specific model production, however, this limits the repeatability potential for trainees to perfect their technique prior to entering the operating theatre, or the opportunity to trial different methods of fixation for the same fracture to determine the best treatment modality [34]. It may result in less need for senior supervision if the areas where more junior colleagues may run into trouble are identified ahead of the operation, thus increasing the capacity for trauma lists.

**Figure 3:**
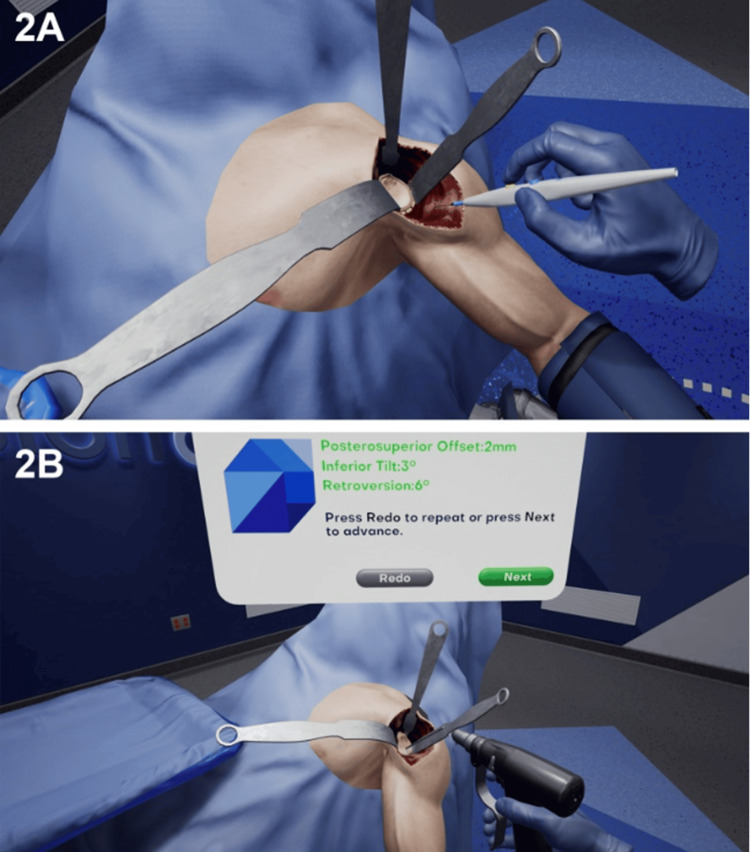
Virtual Reality Simulation Applied to Novel Training Methods Image Source: Lohre et al., 2020 [16]; Open access under the CC BY-NC-ND/4.0 license

There is scope, however, to optimise training from CAVST. The orthopaedic field is seeing a vigorous interest in immersive virtual reality simulation (Figure 3) [35] and the incorporation of simulated haptics has further increased the fidelity of this training experience with potential for application to patient-specific anatomy, as has been trialled already in the urology field [36].

Direction for future improvement

The obvious case for improvement in 3D printing is reducing its cost. As the caseload of in-house 3D printing increases, the fixed costs of machinery and bulk buying materials decreases. Progressing to in-house printing (4.5 hours) as opposed to industry printing (five days) [32] would decrease the length of inpatient stays and further reduce costs in the long term.

Regarding CAVST, aside from the incorporation of virtual reality and haptics as stated above, there is scope to implement artificial intelligence to allow for the automatic planning of surgical maneuvers, which can then be utilised with robotic assistance [17]. This represents benefit both in training and in determining the optimal surgical outcome; although, like with any machine learning solution, it is likely to be met with some amount of controversy and defiance. 

## Conclusions

Preoperative planning in complex fractures such as the PHF is imperative to supporting optimal surgical outcomes and plays a strong role in the training of surgeons. The increasing research into CAVST and 3D printing technology is a promising step forward in maximising the potential of this most critical task. While, in regard to routine practice, they are still in their infancy, the multimodal benefits of enhancing preoperative planning, customising implants, enhancing junior training, and improving patient communication are a strong indication for the creation of large multi-centre RCTs.
